# Preparation of Aluminum Nanomesh Thin Films from an Anodic Aluminum Oxide Template as Transparent Conductive Electrodes

**DOI:** 10.1038/srep20114

**Published:** 2016-02-02

**Authors:** Yiwen Li, Yulong Chen, Mingxia Qiu, Hongyu Yu, Xinhai Zhang, Xiao Wei Sun, Rui Chen

**Affiliations:** 1 Department of Electrical and Electronic Engineering, South University of Science and Technology of China, Shenzhen, Guangdong 518055, P. R. China; 2 LUMINOUS! Center of Excellence for Semiconductor Lighting and Displays, School of Electrical and Electronic Engineering, Nanyang Technological University, 50 Nanyang Avenue, 639798, Singapore

## Abstract

We have employed anodic aluminum oxide as a template to prepare ultrathin, transparent, and conducting Al films with a unique nanomesh structure for transparent conductive electrodes. The anodic aluminum oxide template is obtained through direct anodization of a sputtered Al layer on a glass substrate, and subsequent wet etching creates the nanomesh metallic film. The optical and conductive properties are greatly influenced by experimental conditions. By tuning the anodizing time, transparent electrodes with appropriate optical transmittance and sheet resistance have been obtained. The results demonstrate that our proposed strategy can serve as a potential method to fabricate low-cost TCEs to replace conventional indium tin oxide materials.

Recently, transparent conductive electrodes (TCEs) have been studied extensively because of their potential application for next-generation devices such as liquid crystal displays, thin film solar cells, touch screens, and flexible displays^1^,^2^. TCEs are the vital component for these devices, which require high optical transparency with low sheet resistance. Currently, indium tin oxide (ITO), a commonly doped metal oxide, has dominated the main market for TCEs by exhibiting superior performance compared with other electrode materials. However, for third-generation optoelectronic devices, TCEs should be low-cost and flexible, in addition to their good conductive and transparent properties. It is known that ITO suffers from certain problems that limit its further use, such as scarce supply, leading to its high price, and its ceramic nature, which results in brittle and easily damaged films. As a result, alternative materials have been widely developed for the emerging market of future devices^2^.

Research in the previous decade has shown that many potential candidates have the possibility to replace ITO. These prospective materials include thin metal films or grids, metallic nanowires, carbon nanotubes, and graphene. Although carbon materials can lower costs by using solution-based processes, challenges such as high resistance, which commonly exceeds several hundred ohms per square (denoted Ω/sq), still remain^3^,^4^,^5^,^6^. Metallic nanowires can be prepared through a facile solution process, achieving 20 Ω/sq with an optical transmittance of 80%^7^,^8^,^9^. However, this only applies for noble metals, such as silver or gold; in addition, surface roughness is still a concern for practical application. Thus, it is necessary to explore cheaper metal nanostructures with excellent optical and electrical properties as alternative TCE candidates.

Compared with the noble metals, aluminum (Al) seems a suitable alternative because of its low resistivity (*ρ* = 3 × 10^−8^ Ωcm) and abundance. It is known that metal Al is opaque because of the plasma reflection of free electrons^10^,^11^. This effect could be weakened in a nanomesh structure because the oscillation of free electrons could be stopped when they encounter nanopores. Therefore, an Al mesh nanostructure can decrease light reflection, increasing the transmission of incident light. Based on this idea, previous reports have demonstrated the fabrication of metallic Al nanostructures through electron beam lithography or nanosphere lithography^12^,^13^. However, the fabrication process suffers the drawbacks of laborious procedures or a relatively high cost for large-area electrode production. Moreover, the optical transmittance of an Al nanomesh still needs to be improved for practical application^13^. Aluminum can be prepared to form porous alumina structures by anodizing Al directly^14^,^15^. An anodic aluminum oxide (AAO) template has been widely used to synthesize nanostructure materials, especially nanowires and nanotubes^16^,^17^. Furthermore, the preparation of Au, Pt, and graphene nanomesh two-dimensional thin films have been reported from AAO templates, focusing on applications such as flexible electronic devices and field-effect transistors^18^,^19^,^20^. However, with respect to a cheaper Al nanomesh such as a TCE, there is still no relevant research reported.

In this work, we propose a two-step approach to prepare Al nanomesh thin films through direct AAO wet-etching treatment. The porous alumina films derived from anodizing can served as a template to fabricate the Al thin film with a nanomesh structure. The influence of anodizing time on the morphology and optical properties of the films has been characterized and investigated, and the possible mechanism is discussed in detail. It is interesting to note that the proposed strategy avoids the sophisticated microfabrication processes other methods rely on, such as lithography^21^. The electrochemical approach provides a platform for further research regarding Al nanomesh TCEs.

## Results

**Figure 1:**
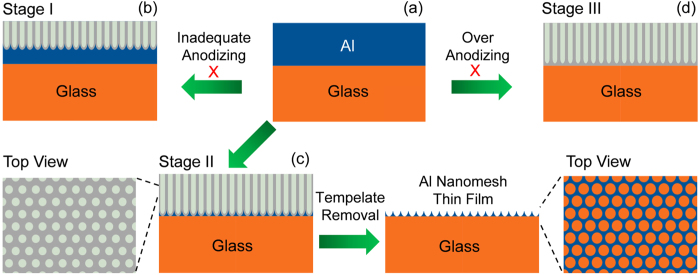
Schematic diagrams show the three different stages during anodizing and etching process for preparing Al nanomesh thin films on glass substrate.

Figure 1 schematically shows the whole fabrication process of the Al nanomesh structure thin film by anodization. As can be seen, with inadequate anodization, there will be Al film remaining on the substrate, sandwiched between the glass and AAO layer (Stage I, shown in Fig. 1b). However, for the case of over-anodization, all of the Al will be oxidized (Stage III in Fig. 1d). Therefore, careful control of the anodization time is essential to alter the amount of remaining Al. As shown in Fig. 1b, with a proper anodizing time, it is possible to achieve Al nanostructures without full oxidation on the substrate. During the subsequent template removal process, a mixed solution of phosphoric and chromic acids can be used to etch the AAO membrane effectively without damaging the Al nanostructures. This strategy has been applied widely for fabrication using the two-step anodization of AAO. The purpose is to obtain ordered porous alumina, and the detailed process can be found elsewhere^14^. The final structure of Al is consistent with the porous AAO template because of the complete etching of alumina walls and barrier layers.

**Figure 2:**
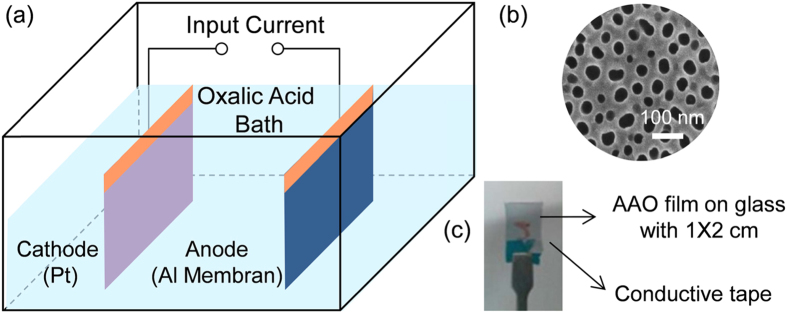
(**a**) Schematic diagram of experimental apparatus. (**b**) AAO thin films onto glass substrate after anodizing. (**c**) The transparent Al nanomesh thin film.

The electrochemical cell for AAO membrane fabrication is shown in Fig. 2a. The complete transformation of aluminum to porous AAO can be identified by monitoring the color of the samples. The AAO membrane products exhibit apparent color change from opaque to transparent (as shown in Fig. 2c), which were totally different from the sputtered aluminum on the glass substrate before anodization. The evident color change proves that a uniform AAO membrane was obtained.

**Figure 3:**
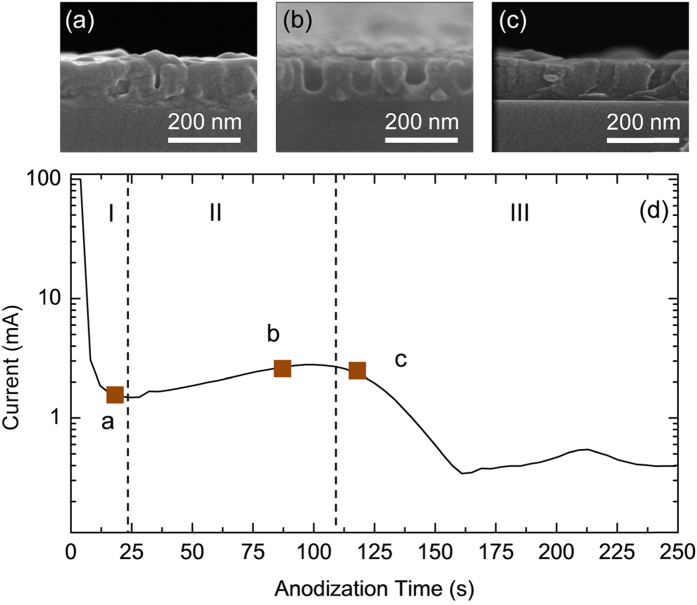
(**a–c**) are the cross-sectional SEM images of Al layer anodizing after 16 s (**a**), 80s (**b**) and 112 s (**c**), respectively. (**d**) Current vs time plot for whole anodizing process of aluminum film in 0.3 M oxalic acid.

During the anodizing process, the correlation between current variation and anodizing time was monitored by a Keithley 2400 recorder. The plot is shown in Fig. 3d and is consistent with the previous report^15^. As shown in Fig. 3, three different stages can be clearly classified. In Stage I, the anodizing current decreased greatly, to 1.49 mA at *t* = 24 s. This can be attributed to anodizing in the Al layer, where porous alumina started to form. The sample was still opaque in this stage. Stage II indicates the stable formation of porous AAO. The ions could migrate under the constant anodizing voltage, resulting in the transport of Al ions outward and O ions inward at the interface on the bottom of pores. The continuous formation of Al ions contributed to the anodic current, and the result can be observed from the slight increase in the current in stage II (the current reached as high as 2.79 mA at *t* = 96 s). For stage III, the current drop means that the anodizing tended to end because the oxidation reaction had reached the interface between Al and glass, finally leading to the complete oxidation of Al. As a result, the current dropped to zero gradually^22^,^23^.

To obtain Al nanomesh thin films from AAO etching, it is crucial to control the remaining sputtered Al nanostructure that has not been anodized below the porous alumina. Figure 3a–c shows the cross-sectional SEM images of porous AAO at specific anodizing times, corresponding to square points in three different stages. First, complete transformation of anodic alumina should not be feasible because the remaining Al is essential for transparent electrodes after alumina etching. Therefore, anodizing termination in Stage III can be excluded, which could be attributed to over-anodizing, leading to full oxidation without any remaining Al layer. The cross-section SEM image in this stage (ending at 112 s) is shown in Fig. 3c. The sample would be expected to be insulating after oxide removal. Alternatively, Stage I could also not be available because the porous structure is still immature, leading to a failure to form the Al nanomesh structure after wet etching. The SEM image in this time range (ending at 16 s) is shown in Fig. 3a. Therefore, only samples in Stage II conditions (shown in Fig. 3b) were selected for the following wet-etching treatment because of the existence of appropriate Al nanostructures during the anodizing process.

**Figure 4:**
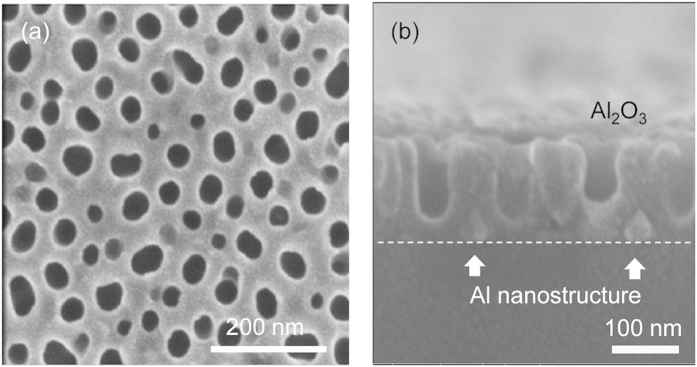
(**a**) SEM images of the surface of AAO membrane. (**b**) Cross-sectional AAO terminated at 80 s, where remaining triangle Al nanostructures are indicated by white arrows.

Combined with the top-view SEM characterization image (Fig. 4a), it is noted that irregular pores with various diameter were formed during the anodizing^15^,^22^. The diameter of the random pores is in the range of 30–50 nm. From the cross-sectional image (Fig. 4b), we can see clearly that the AAO membrane terminated in Stage II at *t* = 80 s, at which time the anodizing nearly reached the bottom of the Al layers while a small amount of metal Al nanostructures remained. The triangles indicated by the white arrows just above the substrate were the remaining Al, as mentioned before^22^. Moreover, there is no similar region for the samples in Stage III (Fig. 3c), which suggests the complete anodizing of all of the sputtered Al. The remaining Al provides the precursor for successful Al TCE preparation following AAO etching treatment.

The subsequent wet-etching process is easy to manipulate. We used phosphoric and chromic mixed acid with a ratio of 6% and 1.8% wt as an etching solution. During the etching time of 40 minutes, no conductivity of the samples was detected, which can be attributed to the compact insulate porous alumina layers covering the remaining Al. Once the alumina were completely removed with an etching time of more than 40 minutes, sheet resistance of thin films in a range of 30 ~ 5000Ω/sq, depending on the terminated anodizing time, could be obtained. However, there was no apparent change in resistance with a further increase in etching time, which implies that there was no reaction between the mixed acid and the remaining Al metal layers. The final electrical and optical properties of the nanomesh are proven to be only affected by the anodizing process, not related at all to the wet-etching process.

**Figure 5:**
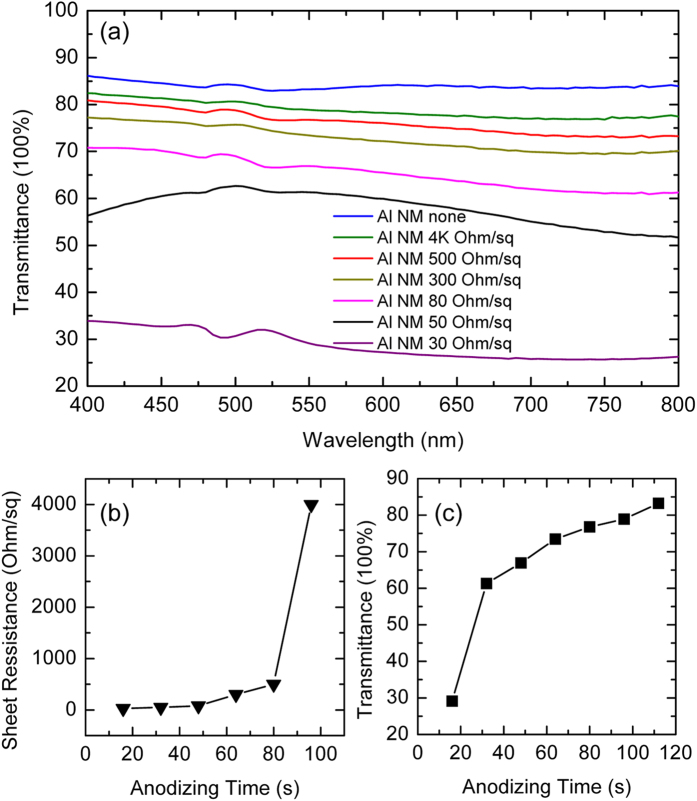
(**a**) Optical transmittance of Al nanomesh thin films with different sheet resistance. (**b**) The sheet resistance of the created film terminated at different anodizing time, and their transmittance at 550 nm (**c**).

Figure 5a shows a summary of various Al nanomesh samples, with their respective sheet resistances and optical transmittances. Sample **1** was terminated in Stage I of anodizing at 16 s, and sample **7** was terminated in Stage III at 112 s. Other samples were in Stage II with times of 32, 48, 64, 80, and 96 s, respectively corresponding to samples **2**–**6**. With an increase in anodizing time, the sheet resistances of the Al nanomesh increased because of smaller amount of remaining Al, ranging from 30 to 4000 Ω/sq for samples **1** to **6**, as shown in Fig. 5b. However, the transmittance of the Al nanomesh thin films shows a continuous increase with anodizing time, as indicated in Fig. 5c. Evidently, samples with larger spectral transmittance suffer from larger sheet resistance. Sample **1** shows the best conductivity, with a resistance that reaches 30 Ω/sq. However, the thin film only shows 28.7% optical transmittance at an incident wavelength of 550 nm. Sample **7** shows a thin film with average transmittances above 85% in the visible light region and 83.4% at 550 nm wavelength, but with a failure to detect the effective conductivity, which indicates the sheet resistance would exceed 10  MΩ/sq, according to the measured system. The others samples in Stage II are proven to have feasible electrical and optical properties for possible application as transparent electrodes. Their transmittances at 550 nm are shown to be 61.3, 66.9, 73.5, 76.8, and 78.9% with increasing anodizing time, corresponding to samples **2**–**6**.

A comparison of resistance and optical transmittance from various TCE materials is shown in Table 1. For the work presented herein, the average optical transmittance was above 70%, which shows a dramatic improvement compared with previous results using the same material (only 55% for an Al nanomesh electrode fabricated by nanopatterning using self-assembled nanoparticles)^13^. In our work, tuned anodizing time could control the remaining Al content, which affects the optical and electrical behavior of TCE thin films directly. However, there is still room for further improvement of the performance by carefully adjusting the space and shape of the Al nanostructure.

## Discussion

We present a facile preparation process for aluminum nanomesh thin films using porous anodic alumina films directly. The AAO was prepared by anodization of a 200-nm-thick sputtered aluminum layer deposited onto a glass slide substrate. SEM characterization as well as transmittance and resistance measurements indicated that the optical and electrical properties of the Al nanomesh corresponded to the termination time during anodizing process. The remaining aluminum layer was vital for TCE performance. The best nanomesh transparent electrodes could achieve 80 Ω/sq and above 70% optical transmittance by taking advantage of their unique structure. We believe that transparent electrodes based on an aluminum nanomesh could be promising for next-generation energy optoelectronic devices.

## Methods

An Al layer with a thickness of approximately 200 nm was deposited on a 1 × 2 cm glass substrate by radio-frequency (RF) magnetron sputtering in a vacuum chamber. The metal layer was deposited with several cycles under a base chamber pressure of 5 × 10^−6^ Torr. The anodizing apparatus is shown in Fig. 2, where constant voltage was supplied by a Keithley voltage supply with model number 2400. Before anodizing, all the samples were rinsed in acetone three times. The Al was anodized in 0.3 M oxalic acid solution with an anodizing voltage of 40 V at 273 K. The whole anodizing process was stopped until the anodizing current remained stable after dropping down. To avoid interface erosion, which would cause the reaction to cease, a tape coating on the interface region between air and solution was used. This approach ensured electrical contact through the Al layer. AAO membranes were obtained after anodizing, along with the conversion of opaque aluminum to transparent alumina. The subsequent wet-etching treatment was carried out in a mixed acid solution of 6% wt phosphoric acid and 1.8% wt chromic acid at room temperature. After AAO template etching, the samples were rinsed by ethanol and deionized water before 10 minutes of drying.

Morphology characterization was performed by scanning electron microscope (Nova NanoSEM 450). Samples were characterized before and after the etching process through top-view and side-view measurements. All samples were sputtered with a thin film of gold to avoid charging effects. The optical properties of the Al nanomesh structure were characterized by a UV-vis spectrometer (PerkinElmer lambda 25), while sheet resistance measurements were performed by a sheet resistance meter with the four-point probe method.

## Additional Information

**How to cite this article**: Li, Y. *et al*. Preparation of Aluminum Nanomesh Thin Films from an Anodic Aluminum Oxide Template as Transparent Conductive Electrodes. *Sci. Rep*. **6**, 20114; doi: 10.1038/srep20114 (2016).

## Figures and Tables

**Table 1:** The comparison of various TCEs materials.

Materials	Methods	Rs (Ω/sq)	Transmittance (100%)	Reference
ITO nanowire	Epitaxial	6.4	80	24
single-wall nanotube	Filtration	30	78	4
single-wall nanotube	CVD	50	70	25
Graphene	CVD	125	97	26
Graphene	Spin Coating	100	85	27
Ag nanowire	Drop Cast	16	86	7
Ag nanowire	Meyer Rod Coat	20	80	28
Cu/Ti	Suptter	16	86	29
PEDOT	Spin Coating	200	80	30
Al nano mesh	Lithography	N.A.	55	13
Al nano mesh	Electrochemistry	80	70	This work
